# HPRT1 Deficiency Induces Alteration of Mitochondrial Energy Metabolism in the Brain

**DOI:** 10.1007/s12035-023-03266-2

**Published:** 2023-02-21

**Authors:** Andrey Y. Vinokurov, Vladislav O. Soldatov, Evgenia S. Seregina, Angelina I. Dolgikh, Pavel A. Tagunov, Andrey V. Dunaev, Marina Y. Skorkina, Alexey V. Deykin, Andrey Y. Abramov

**Affiliations:** 1 Cell Physiology & Pathology Laboratory of R&D Center of Biomedical Photonics, Orel State University, Orel, 302026 Russia; 2 Laboratory of Genome Editing for Biomedicine and Animal Health, Belgorod State National Research University, Belgorod, 308015 Russia; 3 Department of Biochemistry, Belgorod State National Research University, Belgorod, 308015 Russia; 4 Department of Clinical and Movement Neurosciences, UCL Queen Square Institute of Neurology, Queen Square, London, WC1N 3BG UK

**Keywords:** HPRT deficiency, Mitochondrial dysfunction, Complex I inhibition, Reactive oxygen species

## Abstract

Alterations in function of hypoxanthine guanine phosphoribosyl transferase (HPRT), one of the major enzymes involved in purine nucleotide exchange, lead to overproduction of uric acid and produce various symptoms of Lesch-Nyhan syndrome (LNS). One of the hallmarks of LNS is maximal expression of HPRT in the central nervous system with the highest activity of this enzyme in the midbrain and basal ganglia. However, the nature of neurological symptoms has yet to be clarified in details. Here, we studied whether HPRT1 deficiency changes mitochondrial energy metabolism and redox balance in murine neurons from the cortex and midbrain. We found that HPRT1 deficiency inhibits complex I-dependent mitochondrial respiration resulting in increased levels of mitochondrial NADH, reduction of the mitochondrial membrane potential, and increased rate of reactive oxygen species (ROS) production in mitochondria and cytosol. However, increased ROS production did not induce oxidative stress and did not decrease the level of endogenous antioxidant glutathione (GSH). Thus, disruption of mitochondrial energy metabolism but not oxidative stress could play a role of potential trigger of brain pathology in LNS.

## Introduction

Metabolism of purines is vitally important for all living organisms. Purine nucleotides are predominantly synthesized via purine salvage [1], where one of the key enzymes, hypoxanthine–guanine phosphoribosyl transferase (HPRT) (EC.2.4.2.8), is responsible for the production of inosine monophosphate from hypoxanthine and guanosine monophosphate from guanine [2, 3]. Deficiency of HPRT is associated with overproduction of uric acid (leading to arthritis, gouty tophus, and nephrolithiasis) and neuropathology (mental retardation, spastic cerebral palsy, choreoathetosis, and self-injurious behavior) and hematological symptoms (megaloblastic anemia) [4]. The severity of the pathology depends on the enzyme activity which is significantly altered in disease. Complete absence of catalytic HPRT activity manifests itself in the development of all the mentioned symptoms and is classified as Lesch-Nyhan syndrome (LNS) [4, 5]. In the case of partial decrease of enzyme activity (Kelley-Seegmiller syndrome), the main symptom is hyperuricemia, but some neurological conditions can also appear.

LNS is an orphan disease with prevalence between 1/380,000 (in Canada and the USA) and 1/235,000 (in Spain) live births [6]. Furthermore, partial HPRT deficiency is responsible for about 1 in 200 cases of gout [7]. Since the HPRT1 gene is localized on the X chromosome, almost exclusively males develop clinical symptoms whereas heterozygous women are asymptomatic carriers [4].

Although the etiology of symptoms associated with hyperuricemia is relatively well studied, the role of disturbances in the metabolism of purines in the mechanism of development of neurological disorders is not well identified. The levels of HPRT in the CNS are significantly higher than in the rest of the body [8] with the maximal activity of the enzyme in the basal ganglia. Dopamine imbalance is shown for LNS models with the most pronounced effect in the basal ganglia (a 70–90% decrease in the level of dopamine and an increase in the content of serotonin and 5-hydroxyindole acetate) [2, 9].

The mechanism of HPRT-dependent pathology is mostly associated with increased levels of uric acid. Thus, in hepatocytes, the pro-oxidant effect of uric acid leads to a decrease in the ATP level, the activity of succinate dehydrogenase, and cytochrome c oxidase and induces cell death through apoptosis [10]. Uric acid is shown to change mitochondrial metabolism in neurons that leads to cell death [11]. However, interventions reducing uric acid imbalance, even if started at birth, do not attenuate self-mutilation and severe motoric difficulties in LNS patients [5, 12], suggesting independent mechanisms for neurological impairment. Here, we studied energy metabolism and redox homeostasis in primary neurons and astrocytes as well as in acute brain slices and isolated brain mitochondria of CRISPR-ized mice harboring human-like mutation of *Hprt1* gene. We have found that 8Val deletion of the 1^st^ exon of murine *Hprt1* leads to the inhibition of complex I-dependent mitochondrial respiration resulting in decrease in mitochondrial membrane potential that leads to increased production of ROS in mitochondria and cytosol of neurons. However, it did not result in decrease in the level of major endogenous antioxidant—GSH—suggesting that increased production of ROS in neurons expressing mutant *Hprt1* is not enough to induce oxidative stress.

## Material and Methods

### Animals

CRISPR-ized mice with TCG deletion in the first exon (*Hprt1*^del8Val^) serving as a model of LNS have been previously produced in the Institute of Gene Biology of Russian Academy of Science. In brief, mutation detected in a 9-year-old patient suffering from classical LNS was introduced in CBAxC57Bl6J mice with use of CRISPR/Cas9 system [12]. The experimental groups included 2-month-old mice (determination of uric acid level, studies with acute brain slices and oxygen consumption measurements) and 3-day-old pups (source of primary cultures) all in CBAxC57Bl6J background. The groups included *Hprt1*^del8Val^ hemizygous males, *Hprt1*^del8Val^ homozygous females, and WT controls of both sexes. Pups were obtained by breeding of 2-month-old heterozygous *Hprt1*^del8Val^ females with hemizygous *Hprt1*^del8Val^ or WT males. Adult mice were maintained in an air-conditioned room (approximately 20–24 °C) in a 12-h light/12-h dark cycle with free access to food and tap water. All the animal procedures were conducted according to the ARRIVE guidelines and approved by the institutional ethical committee of Orel State University (No. 18 dated 21.02.2020) in compliance with Russian Federation legislation.

### Determination of Uric Acid Levels

≈200 μL of blood was collected in SiO_2_-treated tubes via retro-orbital puncture in mice under slight sedation (Zolazepam 5 mg/100 g, i/p). The isolated brains were weighed and frozen at − 80 °C. Tissue samples of 0.5 g were homogenized in 2.5 mL of 0.9% sodium chloride solution in an ultrasonic bath at 30 kHz for 10 min. The tissue was then placed in a homogenizer for 15 min. The homogenate was centrifuged at 14,000 g for 15 min at + 4 °C. Proteins from supernatant were precipitated with acetonitrile or methanol.

To avoid the effect of “false in vitro elevation of the uric acid level” [13], samples were centrifuged (8000 g for 10 min) as soon as possible after collection. The level of uric acid was determined via uricase/peroxidase method with colorimetric detection [14]. The procedure was performed on biochemical analyzer URIT-880 Vet with the use of commercial kits (Olveks Diagnosticum, Cat #012.012) according to the manufacturer’s protocol.

### Primary Coculture of Neurons and Glial Cells

Cocultures of cortical and midbrain neurons and glial cells were prepared from pups 3 days postpartum as described [13]. After the decapitation cortex and midbrain were removed into an ice-cold Versene solution (Gibco, UK), the tissue was minced and trypsinized (Gibco, Canada) (0.25% for 15 min at 37°C), triturated and plated on polyethylenimine-coated 22 mm coverslips, and cultured in Neurobasal A medium (Gibco, USA) supplemented with B-27 (Gibco, USA) and 2 mM GlutaMax Supplement (Gibco, USA). Cultures were maintained at 37 °C in a humidified atmosphere of 5% CO_2_ and 95% air fed once a week and maintained for a minimum of 12 days before experimental use. Neurons were easily distinguishable from glia: they appeared phase bright, had smooth rounded somata and distinct processes, and laid just above the focal plane of the glial layer. Cells were used at 12–15 days in vitro (DIV) unless otherwise stated.

### Acute Brain Slices

Acute brain slices were prepared according to [14]. After the mouse decapitation, the brain was extracted, removed into a cold HBSS (Gibco, USA) (pH 7.4/4 °C) with subsequent performing a sagittal section and preparation of cortical and midbrain slices with a thickness of 300–500 μm. Slices were kept in a cold HBSS with slight oxygenation. Before the experiments, slices were moved into HBSS (pH 7.4/37 °C) for at least 30 min.

### Oxygen Consumption Measurements

Intact mitochondria were isolated from brains of WT and *Hprt1*^del8Val^ mice by a method of differential centrifugation [15] and resuspended in medium containing 250 mM sucrose (Sigma-Aldrich, Germany), 1 mM EDTA (Sigma-Aldrich, USA), and 19 mM Tris–HCl (Sigma-Aldrich, USA) (pH 7.1/25 °C). Oxygen consumption was measured in a Clark-type oxygen electrode (Hansatech, UK) thermostatically maintained at 25 °C containing 135 mM KCl (Sigma-Aldrich, USA), 10 mM NaCl (Sigma-Aldrich, USA), 20 mM HEPES (Gibco, UK), 0.5 mM KH_2_PO_4_ (Sigma-Aldrich, USA), 1 mM MgCl_2_ (Sigma-Aldrich, USA), and 5 mM EGTA (pH 7.1/25 °C). Glutamate (Sigma-Aldrich, USA) (5 mM) and malate (Acros Organics, Belgium) (5 mM) as mitochondrial complex I substrates were added to allow basal respiration (V2). Data were obtained using an Oxygraph Plus system with Chart recording software. Protein levels were determined using Bradford method with bovine serum albumin as a standard [16].

### Imaging Mitochondrial Membrane Potential

The $\Delta \psi m$ was measured by loading of cells with 25 nM tetramethylrhodamine methyl ester (TMRM) (Invitrogen by Thermo Fisher Scientific, USA) in a HEPES-buffered HBSS for 40 min at room temperature. Measurements were obtained by using a Zeiss 900 CLSM (Carl Zeiss Microscopy GmbH, Jena, Germany) equipped with a × 63 oil immersion objective while keeping 25 nM TMRM in the imaging solution. TMRM was excited using the 561 nm laser line, and fluorescence was measured above 580 nm. Z-stack images were obtained by confocal microscopy, and the basal $\Delta \psi m$ was measured using Zen Blue software (Zeiss). Assessments of the mitochondrial membrane potential maintenance through application of oligomycin (Sigma-Aldrich, USA), rotenone (Sigma-Aldrich, USA), and the uncoupler FCCP (Sigma-Aldrich, USA) were carried out through recordings from a single focal plane [17]. TMRM was used in the redistribution mode to assess the $\Delta \psi m$, and therefore, a reduction in TMRM fluorescence represents mitochondrial depolarization.

### NADH Measurements

NADH autofluorescence in acute brain slices was measured using the setup with a BDLSMN-375 laser source (Becker & Hickl). The excitation radiation through the optical fiber passes through the collimator and then through the beam splitting filter plate, and the lens is directed to the studied area. Emitted fluorescence light was reflected through a 416 nm long-pass filter to a highly sensitive DCC 3260C cooling CCD camera (Thorlabs, USA) [15].

### ROS Assessments

Dihydroethidium (2 μM) (Invitrogen, USA) was used for measurement of cytosolic ROS production. No preincubation (“loading”) was used to limit the intracellular accumulation of oxidized products, and dihydroethidium was presented in the solution during the experiment. Measurements were obtained by using a Zeiss 900 CLSM with a × 63 oil immersion objective. The fluorescence of oxidized form of dihydroethidium was excited by the 561 nm laser line with detection above 575 nm.

For measurement of mitochondrial ROS production, acute brain slices were preincubated with MitoTracker Red CM-H_2_Xros (1 μM) (Invitrogen, USA) for 30 min at 37 °C. Fluorescence intensity measurements were produced using 561 nm excitation and emission above 570 nm [18].

### Glutathione Assessments

Cocultures of neurons and glial cells were incubated with 50 μM monochlorobimane (MCB) (Invitrogen, USA) for 40 min in HEPES-buffered salt solution prior to imaging [19]. Cells were then washed with HEPES-buffered salt solution, and images of the fluorescence of the MCB-GSH were acquired using a Zeiss 900 CLSM with excitation at 405 nm and emission at 435–485 nm.

### Genotyping

Breeders and pups were genotyped with the use of TaqMan system. DNA was extracted from tails via digestion in fresh alkaline lysis buffer (10 mL sterile dWater, 14 μL 50% sodium hydroxide, 14 μL 0.5 M EDTA, pH 8.0) and purification with the use of DNeasy Blood & Tissue Kit (Qiagen, Germany) according to the manufacturer’s protocol. PCR was performed on the CFX-96 (Bio-Rad, USA) platform. The primers used were as follows: F: 5′-CTTTTTGCCGCGAGCCGACC-3′; R: 5′-GCCTGCGTCACTGTGCCAC-3′. TaqMan probes used were as follows: ROX-5′-CAGTCCCAGCGTGGTGAGCCAA-3′-BHQ1 for mutation and FAM-5′-TCCCAGCGTCGTGGTGAGCCAA-3′-BHQ1 for WT allele.

### Statistical Analysis

Data were analyzed with Origin Pro 2018 (MicroCal, Oregon, USA) and are expressed as mean ± SEM. The Mann–Whitney test was used to estimate the statistical significance between experimental groups. Significance was accepted at a 95% confidence level ($p <0.05$). *$p < 0.05$, **$p < 0.001$, and ***$p < 0.0001$.

## Results

### Hprt1 Mutation Results in Increased Levels of Uric Acid

**Fig. 1:**
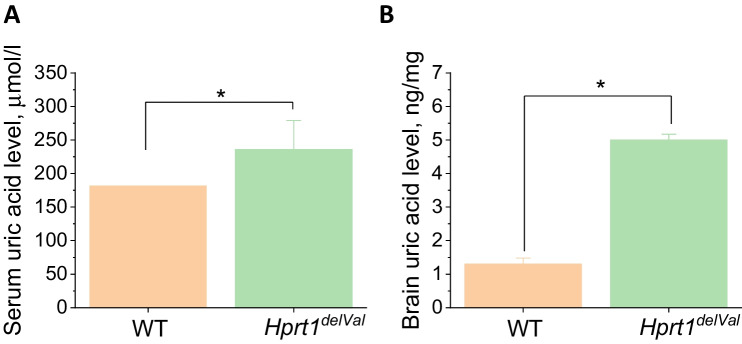
Changes in the level of uric acid in Hprt1-deficient mice. **A** *Hprt1*^*del8Val*^ deficiency increases the level of uric acid in mouse blood serum. Comparative measurements of concentration of uric acid in blood serum of homozygotes and hemizygotes of *Hprt1*^*del8Val*^ and WT controls. **B** Level of uric acid in brain homogenate in *Hprt1*.^*del8Val*^*-*deficient and WT mice. Data are represented as mean ± SEM. *$p < 0.01$

One of the major characteristics of the model efficiency is an increased level of uric acid. *Hprt1*^del8Val^ mice (del8Val/del8Val and del8Val/Y) were characterized by 1.3-fold increased levels of serum uric acid (Fig. 1A). The levels of uric acid were 181.5 [164.0; 186.3] μmol/L and 236 [214.5; 263.3] μmol/L in WT controls ($n=12$) and mutant mice ($n=12$), respectively (Me [Q1; Q3]).

Importantly, *Hprt1*^del8Val^ mice were also characterized by the high level of uric acid in the brain (Fig. 1B). Thus, Hprt1 deficiency induced ~ fourfold increase of the uric acid in brain homogenate (from 1.4 ng/mg in WT to 5.01 ng/mg of tissue; Fig. 1B).

### *Hprt1 Mutation Decreases *$\Delta \psi m$* in Neurons and Astrocytes*

**Fig. 2:**
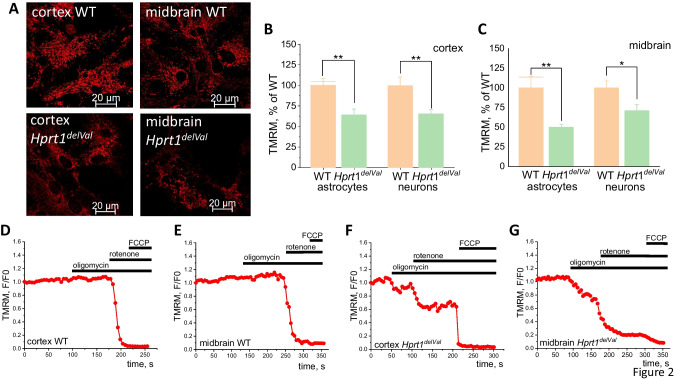
*Hprt1* deficiency decreases mitochondrial membrane potential in neurons and astrocytes and changes the mechanism of $\Delta \psi m$ maintenance. **A** Representative confocal images of cocultures of neurons and glial cells loaded with TMRM, scale bar is 20 μm. **B** $\Delta \psi m$ in brain cells of mutant and WT mice ($n = 5$ coverslips per brain region; $N = 32$–67 astrocytes or neurons per brain region). Data of $\Delta \psi m$ of mutant cells are normalized to TMRM fluorescence intensity of WT astrocytes and neurons. Normalized TMRM fluorescence intensity in WT cortical (**C**) and midbrain (**D**) cells and in *Hprt1*.^*del8Val*^ cortical (**E**) and midbrain (**F**) cells after application of oligomycin (1 μg/mL), rotenone (5 μM), and FCCP (1 μM). Data are represented as mean ± SEM. *$p < 0.05$, **$p < 0.001$, and ***$p < 0.0001$

Mitochondrial membrane potential ($\Delta \psi m$) is an indicator of mitochondrial function, and it can be used to detect possible effect of mutation of the *Hprt1* gene on mitochondrial metabolism. We used TMRM to measure $\Delta \psi m$ in a primary coculture of neurons and astrocytes of WT and mutant mice (Fig. 2A). We have found that cortical and midbrain neurons and astrocytes with *Hprt1* mutation are characterized by lower $\Delta \psi m$ compared to WT astrocytes and neurons (Fig. 2B, C). Thus, the maximal decrease in $\Delta \psi m$ was observed in midbrain astrocytes (by ~ 50%; Fig. 2A, C) with smaller decrease in neighboring neurons (by 29.1%; Fig. 2A, C). In cortical astrocytes and neurons, mitochondrial membrane potential was decreased by 35.9% and 34.7%, respectively (Fig. 2B). Thus, *Hprt1* mutation decreases $\Delta \psi m$ independently of cell regions and cell type.

### *Hprt1 Mutation Changes Mechanism of Maintenance of *$\Delta \psi m$* in Neurons and Astrocytes*

$\Delta \psi m$ Is mainly maintained by the function of electron transport chain (ETC) complexes. Decreased $\Delta \psi m$ in mutant cells may be the result of changes in ETC function due to *Hprt1* functional silencing.

Addition of inhibitor of F0-F1 ATP synthase oligomycin (2 μg/mL) to WT cortical of midbrain neurons and astrocytes exhibits the typical for control lack of effect on $\Delta \psi m$ or induction of small hyperpolarization (Fig. 2D, E). Subsequent application of the inhibitor of the complex I rotenone (3 μM) induced almost complete depolarization in these cells that was confirmed by lack of response to the mitochondrial uncoupler FCCP (2 μM) (Fig. 2D, E). Cortical and midbrain cells with *Hprt1* mutation demonstrate a decrease in TMRM fluorescence intensity after the addition of oligomycin (Fig. 2F, G) that strongly suggests that this enzyme is operating in reverse mode to maintain $\Delta \psi m$ by pumping protons using ATP. It should be noted that in mutant cortical and midbrain cells, rotenone causes only partial decrease of TMRM fluorescence (Fig. 2F, G) compared to control WT cells. Subsequent application of FCCP to these cells induces ~ 20–30% decrease of total TMRM signal that in the absence of any other donor of electrons could be only the activity of complex II. Thus, $\Delta \Psi m$ in cells with *Hprt1* mutation is partially maintained by the complexes II and ATPase due to the complex I reduced function.

### Hprt1 Knockdown Results in Lower Consumption of NADH in Mitochondria

**Fig. 3:**
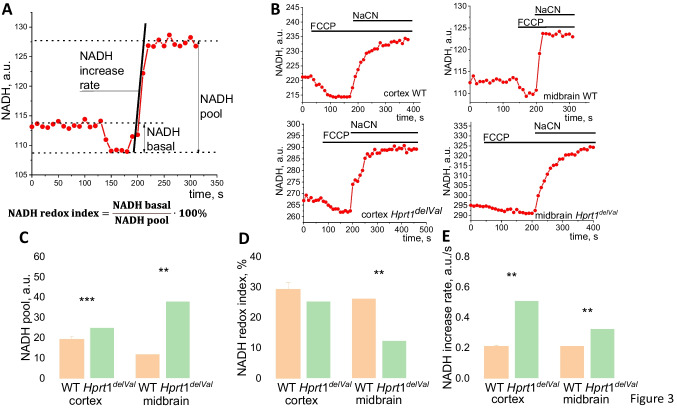
Mitochondrial NADH level and production in *Hprt1-*deficient brain cells. **A** Representative trace of NADH autofluorescence with explanation of calculation of NADH pool, NADH redox index, and NADH production rate. **B** Representative traces of NADH autofluorescence after application of FCCP (1 μM) and NaCN (1 mM). Mitochondrial NADH pool (**C**), NADH redox index (**D**), and NADH production rate (**E**) in acute brain slices of WT and Hprt1 KD mice ($n = 3$ animals, $N = 12$ acute slices of each brain region). Data are represented as mean ± SEM. **$p < 0.001$; ***$p < 0.05$

Mitochondrial metabolism in live cells and in tissues can be also assessed by measurement of mitochondrial redox index. To do this, we measured NADH autofluorescence in acute brain slices from the cortex and midbrain area. To separate mitochondrial NADH from NADPH and nonmitochondrial NADH, protonophore FCCP (to maximize rate of respiration and NADH consumption, taken as 0) and complex IV inhibitor NaCN (to block respiration and consumption of NADH, taken as 100) were used (Fig. 3A, B). These measurements allowed us to assess 3 characteristics which are connected to NADH: mitochondrial NADH content (pool), redox index (ratio between production and consumption of NADH in mitochondria), and the rate of NADH production in the Krebs cycle as determined by the rate of autofluorescence increasing after NaCN (Fig. 3A) [15].

Mitochondrial NADH pool in cortical and midbrain slices from mice with *Hprt1* mutation was 1.3- and 3.2-fold higher compared to that from WT (Fig. 3C). Cortical slices of mutant mice were characterized by an almost similar NADH redox index compared to WT (25.4 ± 2.9% in slices of animals with *Hprt1* mutation and 29.3 ± 2.2% in WT slices), while in the case of midbrain, a significant decrease in NADH redox index was found in *Hprt1*^del8Val^ slices (12.5 ± 1.8% and 26.3 ± 1.7% in mutant and WT animal, respectively) (Fig. 3D). In agreement with higher NADH pool in *Hprt1*^del8Val^ brain, the NADH production rate in the Krebs cycle was significantly higher both in the cortex (in brain slices of mutant mice 0.5 ± 0.04, WT − 0.2 ± 0.01) and in the midbrain (in brain slices of mutant animals 0.3 ± 0.03, WT − 0.2 ± 0.02 (Fig. 3E). Thus, higher level of mitochondrial NADH pool and increased rate of NADH production in mitochondria in brain slices with *Hprt1* mutation suggest that observed in experiments with $\Delta \psi m$ measurements inhibition of complex I activity is not due to limitation of NADH as mitochondrial substrate.

### Hprt1 Knockdown Causes the Inhibition of Complex I-Related Mitochondrial Respiration

**Fig. 4:**
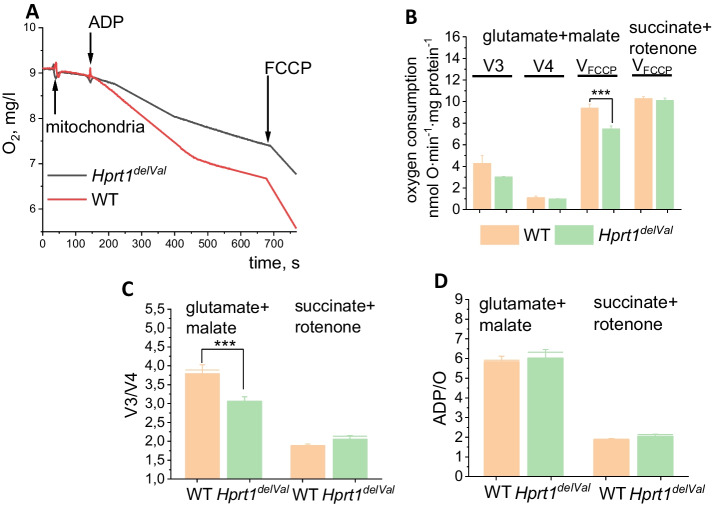
*Hprt1* deficiency induces inhibition of complex I-related respiration of brain mitochondria. **A** Representative traces of ADP-dependent (V3) and ADP-independent (V4) oxygen consumption in brain mitochondria of WT and *Hprt1*-deficient mice; V3, V4, and VFCCP respiration (**B**), respiratory control ratio (V3/V4) (**C**), and ADP/O ratio (**D**) of mitochondria from WT and *Hprt1*.^*del8Val*^ mouse brain in glutamate (5 mM) + malate (5 mM) or succinate (5 mM) + rotenone (5 μM) mediums ($n = 2$ mice, $N = 8$ independent experiments). Data are represented as mean ± SEM. ***$p < 0.05$

Clark electrode was used to estimate the rate of oxygen consumption in isolated brain mitochondria. To assess the parameters of respiration, glutamate (5 mM) and malate (1 mM) were used as substrates of complex I. Brain mitochondria from *Hprt1* mutant mice had a significantly lower rate of ADP-dependent (V3, by 30.2%) and to a lesser degree ADP-independent rate of respiration (Fig. 4A). As a result, the respiratory control ratio (RCR) was decreased by 18.4% (Fig. 4B). The ADP/O in the mitochondria of mutant mice was 7.1 ± 1.1 and in WT mitochondria − 5.0 ± 0.6 (Fig. 4D). Importantly, the maximal rate of the respiration of mitochondria induced by 0.5 μM FCCP was significantly lower in Hprt1 mutant mice compared to control mitochondria that confirm inhibition of the complex I-dependent respiration. In contrast, maximal FCCP-induced respiration of brain mitochondria from *Hprt1*^del8Val^ mice was similar to control in the medium containing 5 mM succinate + rotenone (5 μM) suggesting that the lack of Hprt1 has no effect on complex II activity (Fig. 4B). It should be noted that Hprt1 mutation did not induce changes in respiratory control ratio (Fig. 4C, D) or ADP/O (Fig. 4D) in succinate/rotenone-containing medium.

### *Inhibition of Complex I in Hprt1*^*del8Val*^* Leads to Mitochondrial ROS Production*

**Fig. 5:**
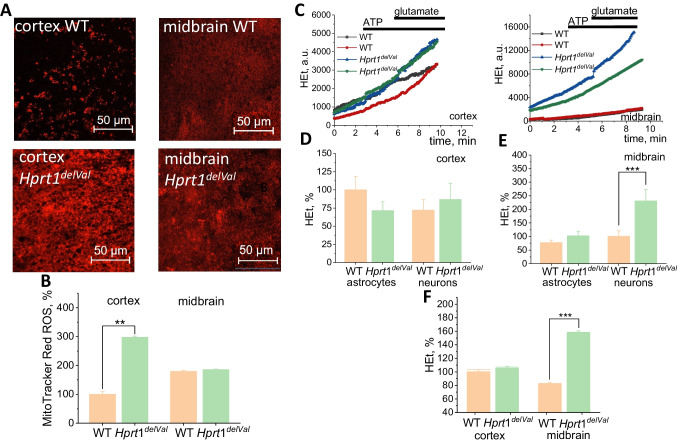
*Hprt1* deficiency results in increase of ROS production in mitochondria and cytosol of brain cells. **A** Representative confocal images of acute cortical and midbrain slices of WT and mutant mice loaded with MitoTracker Red CM-H_2_Xros. Scale is 50 μm. **B** MitoTracker Red ROS fluorescence intensity in acute brain slices, normalized to the level of WT cortical acute slices ($n = 3$ mice, $N = 9$ acute slices of each brain region). **C** Representative traces of HEt fluorescence in cortical and midbrain cells of WT and Htrp1 KO mice. Application of ATP (100 μM) and glutamate (10 μM) was used for differentiation of signal from astrocytes and neurons, respectively. **D, E** HEt fluorescence increase rate in cortical and midbrain astrocytes and neurons of WT and mutant mice normalized to the level of WT cortical astrocytes ($n = 4$ coverslips per brain region; $N = 27$–51 astrocytes or neurons per brain region). **F** Rate of Het fluorescence increase in cortical and midbrain acute slices. Data are represented as mean ± SEM. ***$p < 0.05$

Pharmacological or molecular inhibition of complex I can lead to increase of mitochondrial ROS production [20, 21]. In order to find if inhibition of complex 1 in *Hprt1*^del8Val^ increases mitochondrial ROS production, we used MitoTracker Red ROS as a fluorescent indicator of ROS in this organelle. We have found that *Hprt1* mutation significantly increases the rate of mitochondrial ROS production in primary neurons and astrocytes (Fig. 5A, B). It should be noted that the effect of *Hprt1* mutation on mitochondrial ROS was 3 times higher in cortical neurons and astrocytes in acute brain slices of mutant mice compared to WT acute brain slices (Fig. 5B).

### The Rate of ROS Production Is Increased in Astrocytes and Neurons of Midbrain Cells of Mutant Mice

Mitochondria can produce ROS not only in the matrix but also in the cytosolic side [22] to contribute ROS production from other enzymatic sources. To estimate the rate of ROS formation, a fluorescent probe dihydroethidium (HEt) was used. To differentiate the signal from different cell types in primary cocultures, ATP (for astrocytes) and glutamate (for neurons) were used during the experiment (Fig. 5C). All the data on ROS production rate are compared with the rate of ROS production in WT cortical astrocytes. A reduced level of basal ROS production in astrocytes of mutant mouse cortex was observed (70.7 ± 12.8% of WT cortical astrocytes), while ROS production rate in neurons is slightly increased (86.8 ± 22.2% and 72.1 ± 14.3% in mutant and WT cells, respectively) (Fig. 5D). In the midbrain, an increase in the HEt oxidation rate was found (by 32.0% in astrocytes and 129.9% in neurons compared to WT cells) (Fig. 5E).

In the experiments with acute brain slices, Htrp1^del8Val^ did not change the rate of ROS production in cortex ($N=3$) but increased the rate of HET oxidation in midbrain slices (to 160%, $N=3$) that confirms results obtained with primary midbrain neurons (Fig. 5F).

### High Levels of Reduced Glutathione Protect Cells with Hprt1 Knockdown from Oxidative Stress

**Fig. 6:**
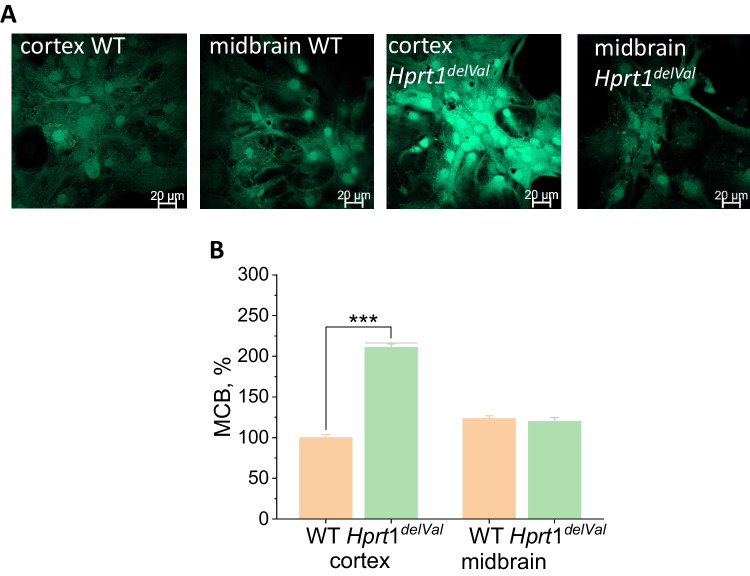
*Hprt1* deficiency increases level of glutathione. **A** Representative confocal images of cocultures of neurons and glial cells loaded with MCB. **B** GSH-MCB fluorescence intensity in brain cells of WT and *Hprt1* mutant mice normalized to the level of WT cortical cells ($n = 6$ coverslips per brain region; $N = 44$–81 cells per brain region). Data are represented as mean ± SEM. **$p < 0.001$

Increased ROS production can lead to the damage of lipids of membranes, proteins, and DNA and the development of oxidative stress [23]. However, this is happening in the case of an imbalance between the production of ROS and its neutralization by the cellular antioxidant system. Glutathione is one of the most important endogenous antioxidants. The glutathione reduced form (GSH) content in the cells was determined using monochlorobimane (MCB), which forms fluorescent conjugates in reaction with GSH (Fig. 6A). The GSH content in cortical cells of mutant mice was more than 2 times higher compared to WT cortical cells taken as 100% (Fig. 5B). There was no statistically significant difference in GSH content between midbrain cells of animals with *Hprt1* mutation and WT midbrain cells (119.9 ± 4.9% and 123.3 ± 3.7%, respectively) (Fig. 6B). Thus, brain cells of mutants are characterized by the balance between ROS production and neutralization and there is no detected oxidative stress.

## Discussion

Here, we demonstrated that HPRT deficiency leads to changes in mitochondrial metabolism that results in decrease in $\Delta \psi m$ of neurons and astrocytes. This changes not only the value of $\Delta \psi m$ but also the mechanism of maintenance of potential from electron transport chain in control to partial compensation of activity of ETC with reverse mode (pumping H + using ATP produced mainly in glycolysis) in F0-F1 ATPase (Fig. 2). It should be noted that this type of compensation of $\Delta \psi m$ in neurons is shown for many pathologies including neurodegeneration and neurons with pathology of complex I [20, 24].

Our data suggests that HPRT deficiency leads to inhibition of mitochondrial complex I that was confirmed in experiments with isolated brain mitochondria (with more pronounced effect in ADP-dependent respiration), lower consumption of the mitochondrial NADH (major substrate and donor of electrons for complex I), and a decrease in $\Delta \psi m$. Although in our experiments Hprtp1 deficiency increased uric acid in brain tissue, this type of mitochondrial pathology has not been shown for pathologies induced by uric acid [25, 26] and we could suggest that mitochondrial dysfunction in neurons and astrocytes with HPRT deficiency is induced by more complex effect rather than changes in intracellular or extracellular uric acid concentration. It can also be confirmed by results obtained from primary neurons and astrocytes which could not be affected by extracellular uric acid.

Interestingly, the rate of NADH production was higher in cells with HPRT deficiency suggesting that inhibition of complex I in these cells is not induced by the lack of substrates and that upstream of glucose metabolism is not disrupted in neurons and astrocytes and may be even higher due to higher glycolytic activity for ATP production.

Similarity in the effects on mitochondria in brain slices from adult mice and in primary neurons and astrocytes prepared from young mice and in isolated mitochondria from adult animals suggests that inhibition of complex I is induced by endogenous processes inside neurons and astrocytes (including alteration of purine biosynthesis or NO generation) but not induced by metabolites (including uric acid) which is produced in tissues other than brain.

The rate of energy metabolism and redox balance varies between brain regions [15, 19]. However, we found more pronounced effect of HPRT deficiency on mitochondria midbrain cells compared to cortical neurons and astrocytes that is in agreement with HPRT pathology associated with the midbrain with much less effect in other brain areas [2, 9]. Considering this, we may suggest that the effect of HPRT on complex I-dependent mitochondrial respiration could be in mitochondria selectively isolated from the midbrain area but not from the total brain that we had.

Mitochondrial ROS overproduction in HPRT-deficient cells is typical for cells with inhibition of complex I [21, 27] and produced in electron transport chain by several potential mechanisms including reverse flow of electrons.

Cytosolic ROS production in HPRT-deficient cortical neurons and astrocytes (Fig. 5) can be potentially associated with enhanced level of xanthine that leads to production of superoxide anion in xanthine oxidase similar to the way how it is produced in neurons in ischemia [28, 29]. Despite relatively high ROS production in mitochondria and cytosol of HPRT-deficient cells, it did not induce decrease in GSH and further oxidative stress. One of the possible explanations of this effect could be the ability of uric acid to scavenge peroxynitrite and possibly superoxide that in some studies led to neuroprotection against hypoxia [30]. It could also be induced by higher levels of NADPH that was shown for HPRT deficiency that can lead to higher GSH synthesis [31]. Thus, disruption in the energy metabolism of midbrain neurons and astrocytes induced by HPRT deficiency potentially can lead to energy deprivation in ATP-consuming conditions (such as an excessive glutamate exposure or ischemia [32]) and be a main trigger for pathology.

Interestingly, despite that HPRT-deficient murine cells were previously shown to have a higher rate of uric acid accumulation than the wild-type cells [33], as far as we know, this is the first report of increased levels of serum uric acid in a genetic model of LNS. Moreover, *Hprt1*^*Del8Val*^ mice are the first murine model of LNS harboring humanized mutation of *Hprt1* gene. Previous bioinformatic analysis revealed that Del8Val mutation could affect homodimerization of the HPRT causing the lack of its enzymatic activity in observed LNS patient. Deletion is found to be located in the HPRT monomer surface close to the dimer junction, and the absence of the aliphatic Valine could be the reason of the hindered monomers interaction. Because of high similarity of human and murine HPRT coding sequence, patient-derived Del8Val mutation was introduced in mouse genome [34]. Computational models show that changes in CRISPR-ized murine HPRT must be similar to that existing in humans [12]. Obviously, in contrast to previously described knockout mice with complete absence of HPRT, this strain may save partial activity of the enzyme resulting in different cellular responses to genetic modification. Previously, it was hypothesized that the reason why mice are resistant to the lack of HPRT activity is the compensatory gain of function of the ARNT, an enzyme performing the same catalytic reactions [35]. Thus, in *Hprt1* knockout mice develop increased level of uric acid only after administration of 9-ethyladenine, ARNT inhibitor. Theoretically, the switch from HPRT to ARNT pathway may be less effective if cellular HPRT level is normal.

## Data Availability

Data will be made available under the reasonable request.
